# Proteomics profiling and machine learning in nusinersen-treated patients with spinal muscular atrophy

**DOI:** 10.1007/s00018-024-05426-6

**Published:** 2024-09-10

**Authors:** Chiara Panicucci, Eray Sahin, Martina Bartolucci, Sara Casalini, Noemi Brolatti, Marina Pedemonte, Serena Baratto, Sara Pintus, Elisa Principi, Adele D’Amico, Marika Pane, Marina Sframeli, Sonia Messina, Emilio Albamonte, Valeria A. Sansone, Eugenio Mercuri, Enrico Bertini, Ugur Sezerman, Andrea Petretto, Claudio Bruno

**Affiliations:** 1 Center of Translational and Experimental Myology, IRCCS Istituto Giannina Gaslini, Via G. Gaslini, 5, I-16147 Genova, Italy; 2 Department of Biostatistics and Bioinformatics, Institute of Health Sciences, Acibadem Mehmet Ali Aydinlar University, Istanbul, Turkey; 3 Core Facilities-Clinical Proteomics and Metabolomics, IRCCS Istituto Giannina Gaslini, Genova, Italy; 4 Pediatric Neurology Unit, IRCCS Istituto Giannina Gaslini, Genova, Italy; 5 Unit of Neuromuscular and Neurodegenerative Disorders, IRCCS Bambino Gesù Children’s Hospital, Rome, Italy; 6 Centro Clinico Nemo, IRCCS Fondazione Policlinico Universitario Agostino Gemelli, Rome, Italy; 7 Department of Neurosciences, University of Messina, Messina, Italy; 8 Neurorehabilitation Unit, Centro Clinico NeMO, University of Milan, Milan, Italy; 9 Department of Biostatistics and Medical Informatics, School of Medicine, Acibadem Mehmet Ali Aydinlar University, Istanbul, Turkey; 10 Department of Neuroscience, Rehabilitation, Ophthalmology, Genetics, Maternal and Child Health- DINOGMI, University of Genova, Genova, Italy

**Keywords:** Spinal muscular atrophy, Proteomics, Cerebrospinal fluid, Biomarkers, Machine learning, Artificial intelligence

## Abstract

**Aim:**

The availability of disease-modifying therapies and newborn screening programs for spinal muscular atrophy (SMA) has generated an urgent need for reliable prognostic biomarkers to classify patients according to disease severity. We aim to identify cerebrospinal fluid (CSF) prognostic protein biomarkers in CSF samples of SMA patients collected at baseline (T0), and to describe proteomic profile changes and biological pathways influenced by nusinersen before the sixth nusinersen infusion (T302).

**Methods:**

In this multicenter retrospective longitudinal study, we employed an untargeted liquid chromatography mass spectrometry (LC-MS)-based proteomic approach on CSF samples collected from 61 SMA patients treated with nusinersen (SMA1 n=19, SMA2 n=19, SMA3 n=23) at T0 at T302. The Random Forest (RF) machine learning algorithm and pathway enrichment analysis were applied for analysis.

**Results:**

The RF algorithm, applied to the protein expression profile of naïve patients, revealed several proteins that could classify the different types of SMA according to their differential abundance at T0. Analysis of changes in proteomic profiles identified a total of 147 differentially expressed proteins after nusinersen treatment in SMA1, 135 in SMA2, and 289 in SMA3.

Overall, nusinersen-induced changes on proteomic profile were consistent with i) common effects observed in allSMA types (i.e. regulation of axonogenesis), and ii) disease severity-specific changes, namely regulation of glucose metabolism in SMA1, of coagulation processes in SMA2, and of complement cascade in SMA3.

**Conclusions:**

This untargeted LC-MS proteomic profiling in the CSF of SMA patients revealed differences in protein expression in naïve patients and showed nusinersen-related modulation in several biological processes after 10 months of treatment. Further confirmatory studies are needed to validate these results in larger number of patients and over abroader timeframe.

**Supplementary Information:**

The online version contains supplementary material available at 10.1007/s00018-024-05426-6.

## Introduction

Spinal muscular atrophy (SMA) is an autosomal recessive neurodegenerative disorder, due to defects in the survival motor neuron (SMN1) gene on chromosome 5, leading to degeneration of motor neurons in the spinal cord, and inducing a progressive muscular hypotonia and weakness [1].

According to the age at onset and the best motor function achieved, SMA has been classified into five main types with pediatric onset (types 1 to 3, from the most severe to the mildest), and two more recently classified phenotypes, type 0 with antenatal onset, and type 5 with adult onset and a mild phenotype [1]. Disease severity inversely correlates with the number of copies of the SMN2 gene [2, 3], the SMN1 paralogue gene, which produces predominantly an alternatively spliced mRNA transcript lacking the exon 7 and encoding for an unstable SMN protein (SMNΔ7) [4].

In the last decade, significant improvements have been made in SMA treatment, resulting in three available therapies, namely the SMN2 splicing-modifiers nusinersen (antisense oligonucleotide administered intrathecally) and risdiplam (small molecule with oral route), which upregulate SMN protein levels [5, 6], and onasemnogene abeparvovec (an adeno-associated virus vector-based gene therapy administered once i.v.) [7]. These treatments have improved life expectancy and quality of life in SMA patients, particularly when started in the pre-symptomatic or early stages of the disease [8, 9].

In this scenario, neonatal screening programs are mandatory to ensure rapid diagnosis and guarantee a prompt therapeutic intervention, making the discovery of novel biomarkers urgently required.

To date, the only available and clinically relevant biomarkers are represented by the SMN2 copy number, alongside with two alternative splicing-modulating variants (rs121909192 and rs1454173648), although not uniquely predictive of disease severity [1]. In addition to genetic biomarkers, neurofilaments (NfL) have shown potentiality as serum and cerebrospinal fluid (CSF) biomarker, but inconsistent results prevent their wide use in the clinical setting, especially in adult patients [10].

In the last few years, omics-based techniques have been applied for biomarkers discovery in CSF samples of nusinersen-treated SMA patients [11–18], providing valuable insights on nusinersen-related modification of CSF proteome and metabolome, and identified putative treatment response biomarkers. However, no prognostic biomarkers in naïve patients have been explored so far.

In the context of omics data generation and analysis, machine learning algorithms demonstrated exceptional potential for managing extensive and multidimensional datasets, serving as fundamental tools to facilitate biomarker discovery across various fields, including neuroscience, but their application in SMA remains unexplored [19–22].

We conducted a multi-center retrospective longitudinal study, using an untargeted liquid-chromatography mass spectroscopy-based proteomic approach on CSF samples collected from a large cohort (*n* = 61) of SMA1, SMA2 and SMA3 patients at baseline (before starting nusinersen treatment, T0), and after 10 months of therapy (before the sixth nusinersen infusion, T302). The primary objective of the study was to identify CSF prognostic biomarkers at baseline, by combining proteomics data and a machine learning algorithm. The secondary objective was to describe changes in the proteomic profile at T302 and identify which biological pathways are influenced by nusinersen, through the application of classical bioinformatics approaches.

## Results

### Study design and population

**Fig. 1:**
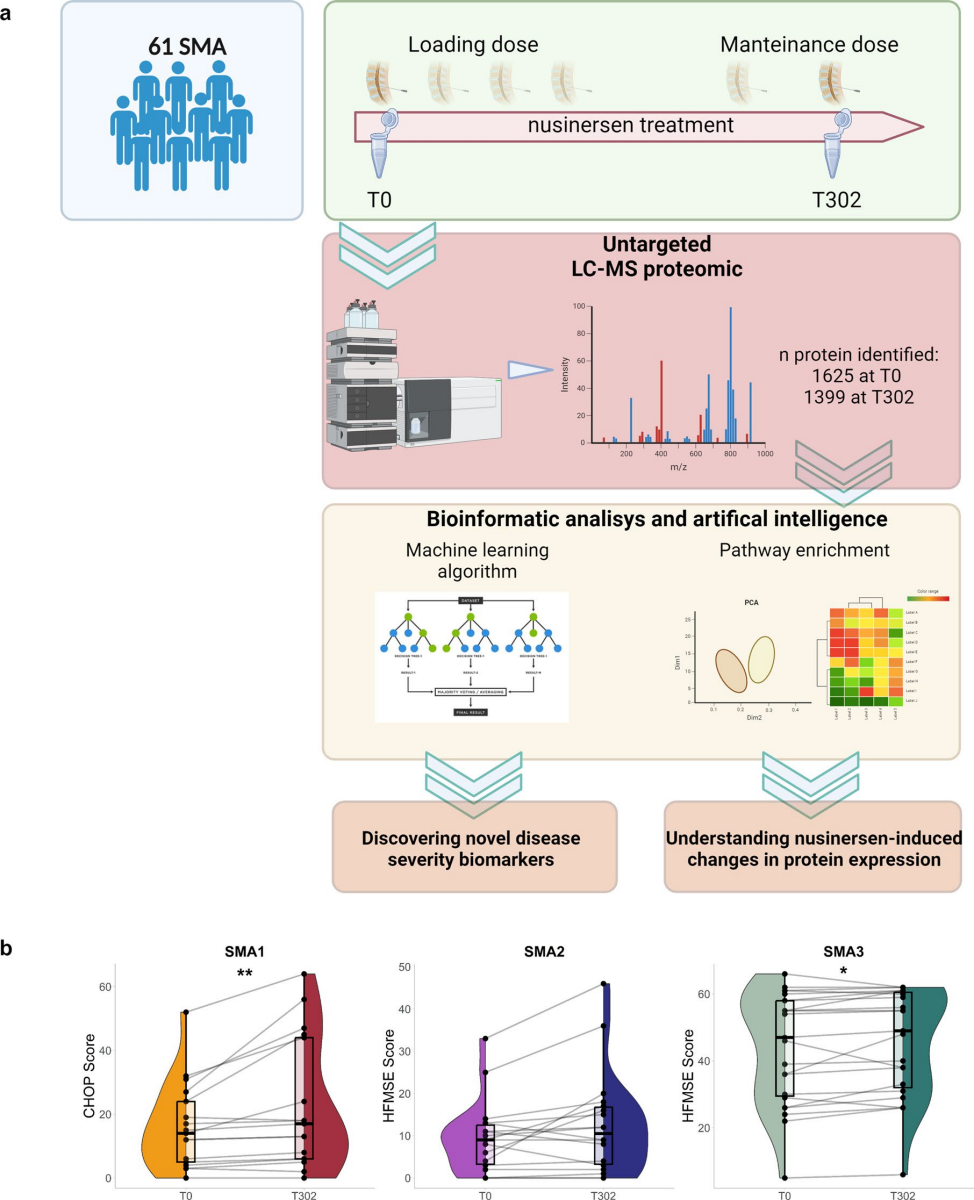
Study design and population. (**a**) A graphical description of the study is shown. (**b**) Changes in CHOP scores for SMA1 (*n* = 17), and HFMSE scores in SMA2 (*n* = 18) or SMA3 (*n* = 23) patients before (T0) and after (T302) nusinersen treatment. Plots show points for each sample score and paired sample data were connected by lines. Violin plot shows the distribution of the scores via density, and box plots showing interquartile range; box has sides belonging to lower and upper quartiles, and median depicted by horizontal line. Significance was tested via paired Wilcoxon rank-sum test. Significance test results were shown by “*“ for p-value < 0.05; “**“ for p-value < .01

The study design is summarized in Fig. 1a.

Sixty-one genetically confirmed SMA patients treated with nusinersen, including 19 SMA1, 19 SMA2, and 23 SMA3 subjects, were enrolled in the study (Fig. 1a). Demographic characteristics and clinical data of patients are reported in Table 1.

**Table 1:** Study population description. Age at baseline, gender, number of SMN2 copies, response to therapy, respiratory support and nutrition information are listed in the table

	SMA1 (*n* = 19)	SMA2 (*n* = 19)	SMA3 (*n* = 23)	Total (*n* = 61)
**Age at T0 (median**,** Q1-Q3)**	2.1 (0.7–4.9)	7.7 (2.3–11.8)	15.3 (7-29.3)	6,9 (2.3–13)
**Gender (F/M)**	11/8	12/7	12/11	30/26
**SMN2 copies (n**,**%)**				
2 SMN2	17 (89%)	2 (10%)	3 (13%)	22 (36%)
3 SMN2	2 (10%)	17 (89%)	10 (43%)	29 (48%)
> 3 SMN2	0	0	9 (39%)	9 (15%)
**Responders (n**,**%)**	11 (52%)	6 (31%)	6 (23%)	23 (37%)
**Respiratory support (n**,**%)**				
Use of non-invasive ventilation	7 (37%)	8 (42%)	1 (4%)	16 (26%)
Tracheostomy	7 (37%)	1 (5%)	0	8 (13%)
**Nutrition (n**,**%)**				
Oral	7 (37%)	19 (100%)	23 (100%)	49 (80%)
Nasogastric tube	8 (42%)	0	0	8 (13%)
Gastrostomy	4 (21%)	0	0	4 (6%)

The median age at T0 was 2.1 years (IQR: 0.7–4.9) in SMA1, 7.7 years (IQR: 2.3–11.8) in SMA2, and 15.3 years (IQR: 7-29.3) in SMA3 (Table 1). Differences in age at T0 were significantly found between the SMA types (SMA1 vs. SMA2, *p* < 0.005; SMA1 vs. SMA3, *p* < 0.0001; SMA2 vs. SMA3, *p* < 0.005; Kruskal-Wallis test, multiple comparison), while gender did not differ among groups (Pearson’s Chi squared test p-value = 0.927).

At baseline, median CHOP values in SMA1 was 13/64 (IQR: 3.7–24), while median HFMSE scores were 9/66 (IQR: 2.7–13) in SMA2 and 47/66 (IQR: 29–58) in SMA3 (Fig. 1b).

### Identification of putative prognostic biomarkers through machine learning

**Fig. 2:**
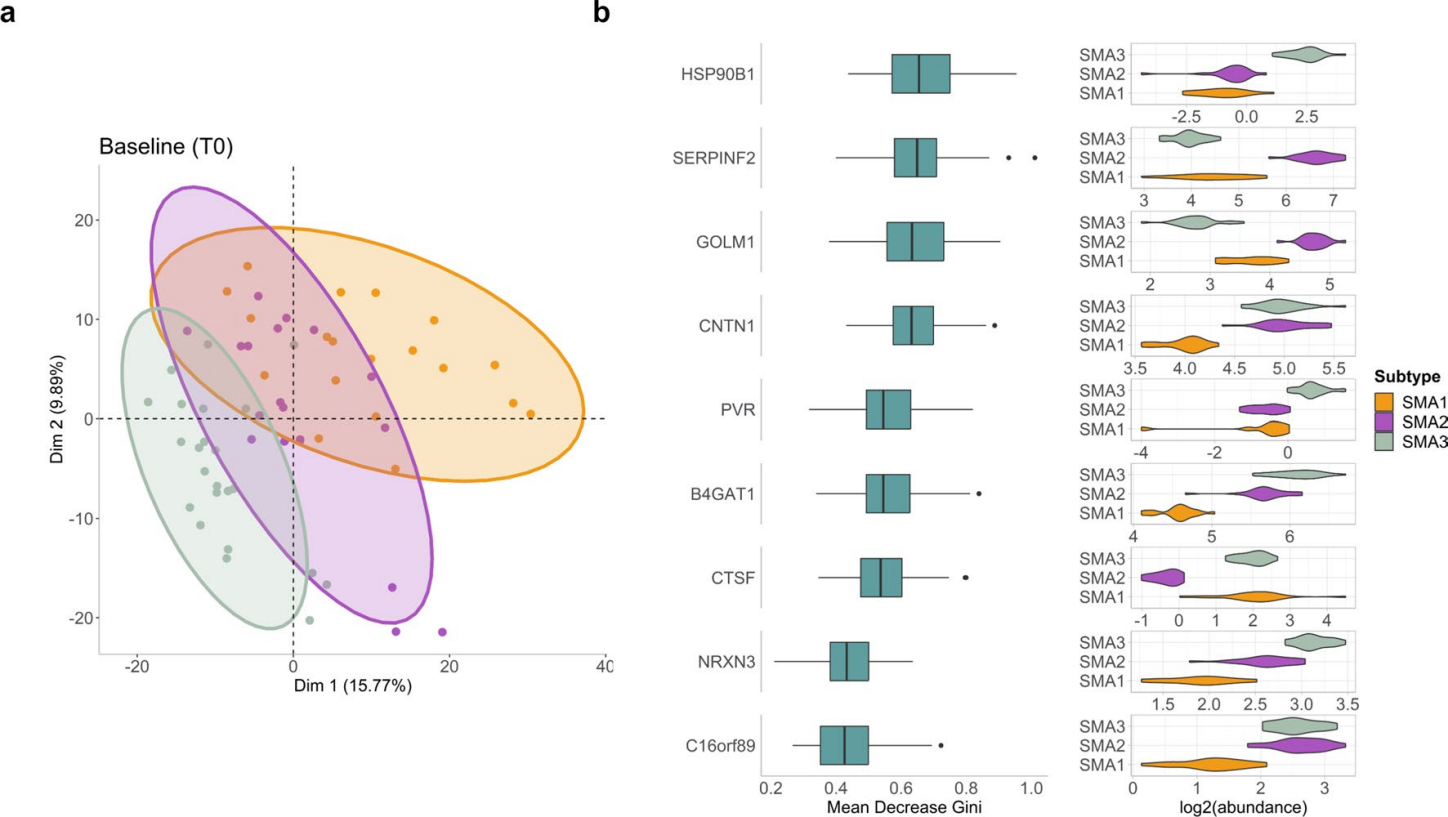
Random Forest identified new putative biomarkers for SMA severity. (**a**) PCA plot showing the separate clustering patterns of SMA1 (orange), SMA2 (purple) and SMA3 (green) proteomic profiles at T0 based on dimensions 1 and 2. On PCA plots, each sample was shown by points, and ellipses correspond to 95% confidence intervals for each of the subtypes. (**b**) Nine proteins common among top 30 features obtained from 100 different Random Forest models for classification of SMA types at T0. On the left side of figure, the importance score (based on mean decrease in Gini index) distributions of each protein from 100 models were illustrated by box plots. Log2 abundance of each protein in each SMA type at T0 were shown by violin plots on the right side. Box plots showing interquartile range, and outliers were shown by black dots

Proteome analysis at T0 identified 1625 unique proteins. Principal component analysis (PCA) shows separation of proteome profiles in the different SMA subtypes (Fig. 2a). PERMANOVA test carried out on Euclidian distance of proteomics data at T0 revealed that only SMA subtype (F = 5.1894, *p* = 0.001) and age (F = 3.4401, *p* = 0.001) can significantly explain the variance in the data, while the rest of the factors, gender, BMI group, SMN2 copy number, and response status, were not significant (*p* > 0.05). Therefore, for the differential abundance analysis, age information was used as a confounding factor.

A machine learning approach, the Random forest (RF) classification algorithm, was then used to test whether the protein expression at T0 could be used to predict the SMA subtypes. All the trained RF models obtained gave accuracy of 1, which is an indicator of over-fitting, and the same accuracy value was obtained from prediction on test datasets, too. To eliminate the possible effects of post-processing of the data and hyper tuning of the RF parameters in model trainings, that might cause data leaking and over-fitting, different strategies were followed, including splitting raw dataset into train and test, and applying normalization procedures independently, changing the ratio of train and test datasets, and varying size of grids for hyper parameter tuning (the detailed strategies employed during optimization and prevention of overfitting and data leakage are described in the Supplementary Methods). All models consistently gave accuracy of 1 on both trained and test datasets, signifying the strength of the proteomics data in SMA subtype classification. Lastly, to further decrease the potential bias of the model on feature selection for the classification arising from the relatively small sample size, 100 different RF models were trained by changing the samples in the train and test datasets, and common proteins across top 30 features, sorted based on mean decrease in Gini coefficient of all 100 RF models, were determined (Supplementary Table 1). Accordingly, 9 proteins were obtained as common features from the 100 models: heat shock protein 90 beta family member 1 (HSP90B1), serpin family F member 2 (SERPINF2), golgi membrane protein 1 (GOLM1), contactin 1 (CNTN1), PVR cell adhesion molecule (PVR), beta-1,4-glucuronyltransferase 1 (B4GAT1), cathepsin F (CTSF), neurexin 3 (NRXN3), and chromosome 16 open reading frame 89 (C16orf89) (Fig. 2b). The maximum number of modes for model training was set to 3, and we observed that the classification of the three SMA subtypes can be achieved using combinations of two proteins selected based on their differential abundance profiles in SMA subtypes. Out of the 9 selected proteins, CNTN1, B4GAT1, NRXN3, and C16orf89 were identified as key proteins that distinctly separate the most severe form SMA1 from the milder ones (Fig. 2b).

We checked the correlations between age and the 9 proteins above (Supplementary Table 1). Except SERPINF2 and CTSF, 7 of them show a significant moderate correlation with age (p.adj < 0.05). Among those, only GOLM1 (rho = -0.45) had negative correlation, while the other 6 proteins, HSP90B1 (rho = 0.61), CNTN1 (rho = 0.48), B4GAT1 (rho = 0.77), PVR (rho = 0.56), NRXN3 (rho = 0.47), and C16orf89 (rho = 0.55) revealed positive associations.

### Nusinersen-driven changes in proteomic profile at T302

**Fig. 3:**
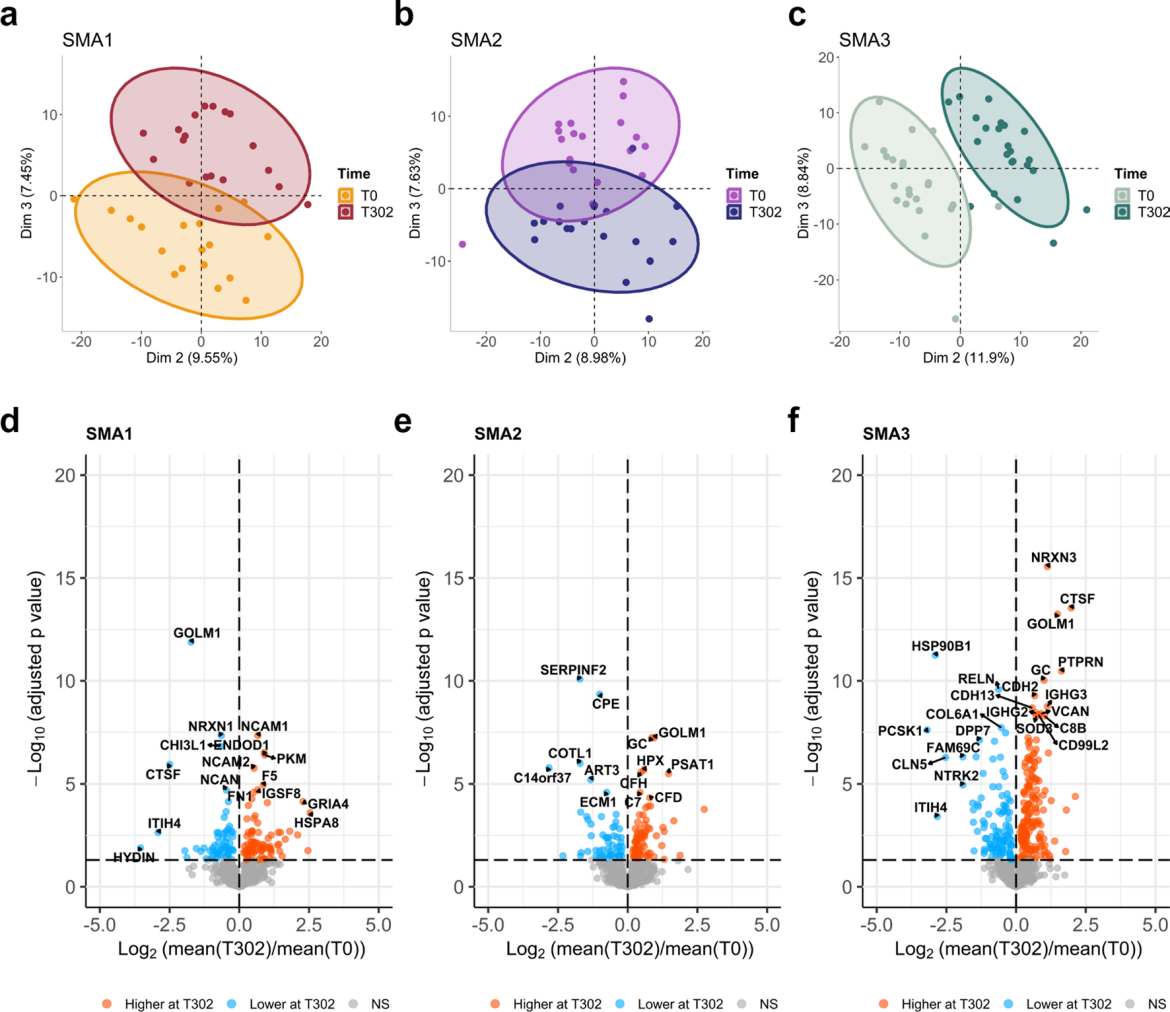
Nusinersen modulates CSF proteome at T302. The change in clustering based on proteomics between T0 and T302 is shown for (**a**) SMA1, (**b**) SMA2, and (**c**) SMA3 patients, based on second- and third-dimension combinations. PCA plots showing distribution and clustering of samples, and ellipses correspond to 95% confidence intervals for each time point. Volcano plots are used to show the down- or up-regulated proteins after nusinersen treatment for (**d**) SMA1, (**e**) SMA2, and (**f**) SMA3 samples. Significantly changed proteins (p.adj < 0.05) were colored on volcano plots based on their higher (orange) or lower (blue) abundances at T302 compared to T0

Proteome analysis at T302 identified 1399 unique proteins. Initially, all samples from each group, independent from their response status, were incorporated into the analysis of protein changes between T0 (before treatment) and T302 (after 302 days of treatment). When PCA plots were generated using different pairs of axes, more distinct separation was observed when examining dimensions other than first two, as PCA results can be influenced by biases originating from any of the preceding steps, starting from data collection to the end of data processing [23] (Fig. 3a-c).

When the significant changes in proteomic profiles in response to nusinersen-treatment were examined between T0 and T302 for each SMA subtype individually, 147, 135, and 289 proteins were founded, changing significantly in SMA1, SMA2, and SMA3, respectively. Volcano plots highlight downregulated and upregulated proteins at T302 compared to the baseline (Fig. 3d-f), and the heatmaps show hierarchical clustering of significantly changing proteins (Suppl. Figure 1a-c).

**Fig. 4:**
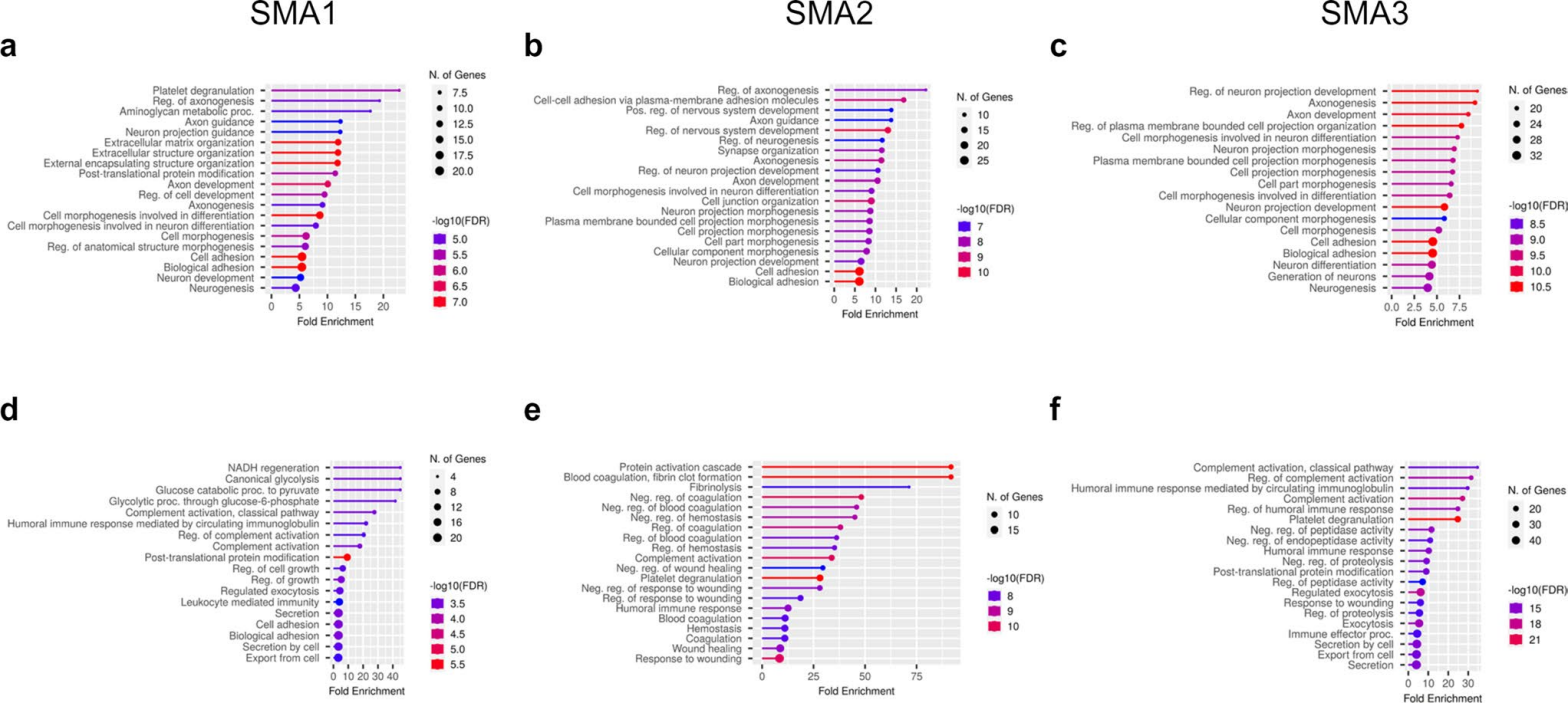
Biological processes modulated by nusinersen at T302. Top 20 GO pathways for biological processes (BP) enriched by mainly down-regulated (**a**-**c**) and up regulated DEPs (**d**-**f**), obtained from two clusters from each heatmap, are shown by lollipop plots for each SMA type. Fold enrichment represents the ratio of the percentage of matched proteins in the query list with pathway-associated proteins to the percentage of query with the background. Bar colors are corresponding to the false discovery rate (FDR) corrected p values for each enriched pathway, and the size of dots are proportional to the number of proteins associated with the respective pathway

Gene ontology (GO) enrichment analysis of biological processes (BP) was performed to investigate enriched pathways of differentially expressed protein (DEPs) after nusinersen treatment. Overall, a common response across all SMA subtypes was observed for proteins down-regulated by the treatment, with the main top enriched pathways related to axonogenesis (GO terms: regulation of axonogenesis, axon guidance, axon development (Fig. 4a-c). Axonogenesis related proteins included components of the semaphorin family (SEMA6A, SEMA7A, SEMA4B), leucine rich repeat and Ig domain containing 1 (LINGO1), plexin-B2 (PLXNB2), L1 cell adhesion molecule (L1CAM), multiple EGF like domains 8 (MEGF8), dystroglycan 1 (DAG1), and reelin (RELN).

Other than the axonogenesis, SMA1 subjects showed platelet degranulation and aminoglycan metabolic processes within the top modulated BPs for down-regulated DEPs.

Pathway enrichment analysis of proteins up-regulated after nusinersen treatment, revealed disease-specific results for SMA1 and a similar signature for the milder phenotypes SMA2 and SMA3 (Fig. 4d-f).

In SMA1 patient’s proteome after treatment, up-regulated DEPs were related to glucose metabolism (GO terms: canonical glycolysis, NADH regeneration, glucose catabolic processes to pyruvate), including proteins as pyruvate kinase (PKM), enolase 1 (ENO1), aldolase, fructose-bisphosphate C (ALDOC), phosphoglyceratemutase 1 (PGAM1).

In SMA2 and SMA3 proteomes, up-regulated DEPs mainly belonged to two highly intertwined cascades, namely the coagulation and the complement pathways. In details, proteome of SMA2 subjects after treatment revealed increased coagulation processes (GO terms: protein coagulation cascade, blood coagulation and fibrinolysis), identified by proteins as fibulin1 (FBLN1), coagulation factor II, IX and XII (F1, F9, F12), kininogen1 (KNG1), fibronectin 1 (FN1), serpin family G member 1 (SERPING1), fibrinogen A (FGA), and apolipoprotein E (APOE).

SMA3 proteome after treatment showed enhanced complement activation (GO terms: regulation of complement activation and humoral immune response), including proteins as complement C1q B and C chain (C1QB, C1QC), complement C8 beta chain (C8B), complement molecules (C2, C3, C4A, C6, C7), complement C1r (C1R), complement C1s (C1S), complement Factor I (CFI), clusterin (CLU), and serpin family G member 1 (SERPING1). Complement activation (GO terms: complement activation, classical pathway) also appeared in SMA1 pathway enrichment but was not as significant for this subtype. Proteins included were C1S, CFI, C2, and C4A.

### Proteome changes in responder versus non-responder patients

In addition to assessing the temporal changes in protein levels for each SMA subtype using all patient samples, we conducted a comparison of protein fold changes between baseline (T0) and after 302 days of treatment (T302) in responder (R) and non-responder (non-R) patients.

**Fig. 5:**
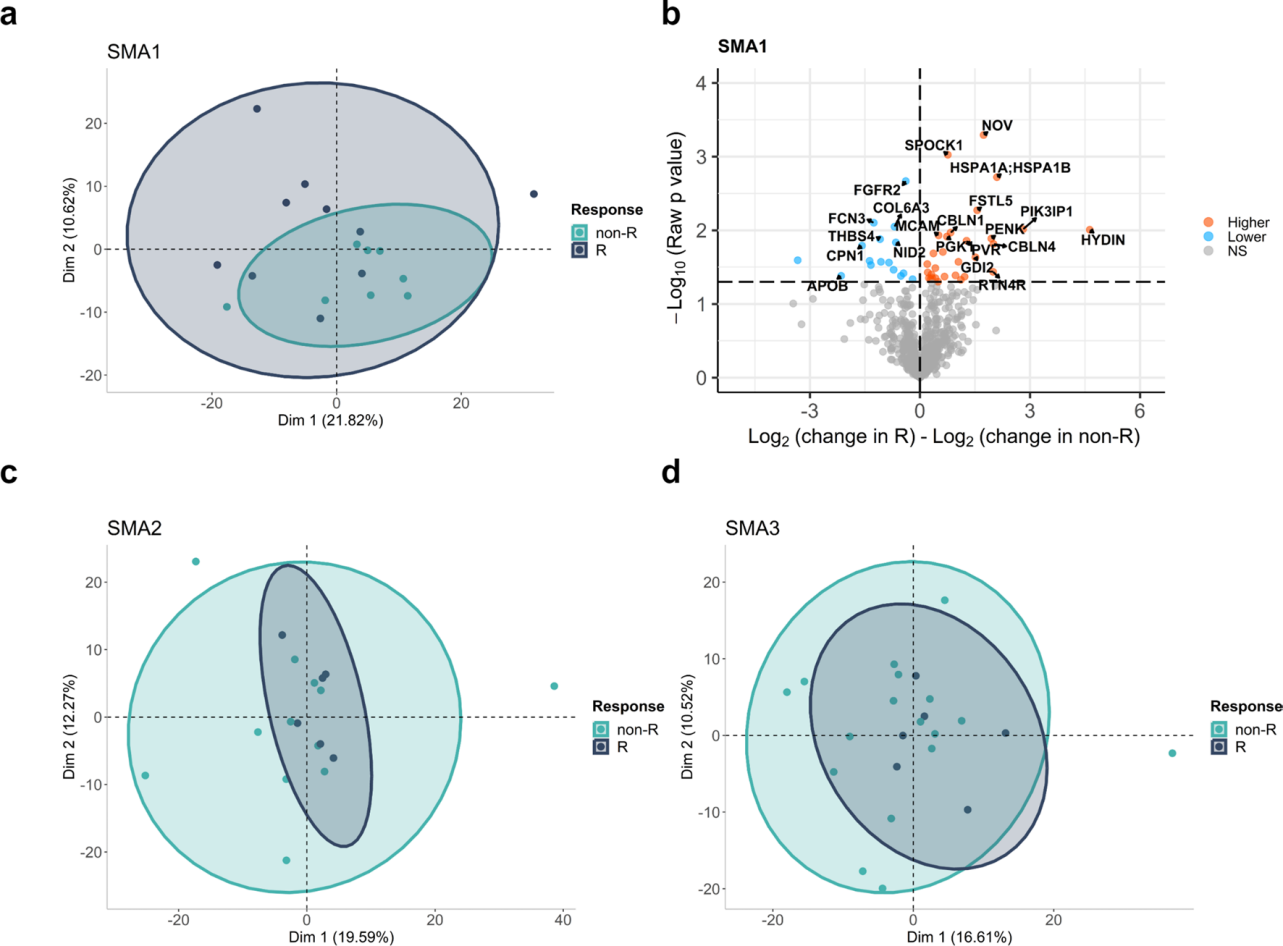
Responders vs. non-Responders analysis. The clustering patterns of the responders (green) and non-responders (dark grey) based on changes in protein levels after nusinersen treatment were shown by PCA plots based on first two dimensions for (**a**) SMA1, (**c**) SMA2, and (**d**) SMA3. Each dot represents one sample, and ellipses correspond to 95% confidence intervals for each of the subtypes. (**b**) Volcano plot for comparison of time-wise changes in protein levels in each responder and non-responder samples. Fold change differences were calculated by subtracting log2 fold change of proteins between T302 and T0 in non-responders from log2 fold change between T302 and T0 in responders, and higher and lower changes in responders compared to non-responders were shown by orange and blue colors, respectively

Based on outcome measures improvement at T302, we classified as responders 52.6% of SMA1, 33.3% of SMA2, and 26.1% of SMA3. When we check the importance of response status in treatment-based protein level changes by PERMANOVA test for each SMA subtype, only SMA1 had a significance close to threshold (F = 1.2544, *p* = 0.085), while the protein changes were determined to be similar between R and non-R in SMA2 (F = 0.8361, *p* = 0.739) and SMA3 (F = 1.0221, *p* = 0.416) groups. Accordingly, principal components analysis showed better separation of treatment-based CSF proteome change between R and non-R patients in the SMA1 group compared to the SMA2 and SMA3 groups (Fig. 5a-c-d).

The comparison of changes in proteome expression between R and Non-R patients identified 45 DEPs in the SMA1 group, 26 in SMA2, and 26 in SMA3. Volcano plot highlighting downregulated and upregulated proteins at T302 in R vs. non-R was therefore depicted for SMA1 only (Fig. 5b). Among proteins upregulated in SMA1 Responders after treatment, we highlight the cellular communication network factor 3 (NOV/CCN3, *p* = 0.0005), involved in immunomodulatory processes via T regulatory cells [24], the SPOCK1 (*p* = 0.00094),an extracellular proteoglycan that belongs to the secreted protein acidic and rich in cysteine (SPARC) family and is involved in the regulation of blood brain barrier (BBB) permeability [25], and the heat shock proteins HSPA1A/HSPA1B (*p* = 0.002), which are highly conserved cellular response to internal and external stress [26]. Fibroblast growth factor receptor 2 (FGFR2, *p* = 0.002), involved in lymphocyte and macrophage/microglia infiltration as well as myelin and axon degeneration [27], was instead in the pool of downregulated proteins.

## Discussion

Advancements in omics technologies have significantly expanded our understanding and exploration of biological mechanisms, facilitating biomarker discovery in neuroscience [28–31]. Machine learning algorithms, a subset of artificial intelligence, prove particularly effective in handling omics datasets, capturing intricate patterns that might be overlooked by traditional statistical methods [19–22, 32, 33].

In the first part of this study, we exploited machine learning to discover novel prognostic biomarkers for SMA. The Random Forest algorithm, applied to the protein expression profile of naïve patients, identified different pivotal proteins capable of classifying the different SMA types based on their differential abundance. Among them, CNTN1 and NRXN3 clearly differentiated the severe form from the milder ones.

CNTN1, one of the axo-glial adhesion molecules, showed the lowest expression levels in SMA1 and higher expression in SMA2 and SMA3 patients. CNTN1 is expressed both in the central and peripheral nervous system [34] and is primarily located at the paranodes, where it forms a tripartite complex contributing to the stabilization of the connection between the axon and the myelin loops [35]. Abnormal intercellular communication between axons and Schwann cells have been recently associated with immature, dysfunctional and vulnerable motor axons in severe SMA patients’ human tissues and in a severe SMA mouse model (SMAΔ7 mice) [36].

Moreover, genetic ablation of Cntn1 in a zebrafish model results in infertility, reduced animal size, ataxic swimming behavior, and a curved spine, with hypomyelinated neurons compared to wild-type [37]. Similarly, the Ctntn1-/- mice, display failure to thrive and severe locomotor abnormalities, along with a decreased number of myelinated axons in both the spinal cord and the optic nerve [37]. In the Smn1-/-Smn2+/- mouse model, a transcriptomic analysis identified the tnfa-il6-cntn1 pathway as one of the top upregulated pathways in the central nervous system, where Cntn1 might serve as a protective factor for neurons [38]. Together, these findings point to a crucial role of CNTN1 in the developing nervous system, with possible implications in the pathogenesis of SMA.

NRXN3, a member of the neurexin family (NRXN1, 2 and 3), presynaptic cell adhesion molecules necessary for synapse formation and maintenance [39–41], exhibited a progressive increase in expression levels, with the lowest levels observed in SMA1 patients and the highest in SMA3 patients at baseline. Interestingly, a reduction or altered Nrxn2 splicing have been observed in both transgenic SMA mice and zebrafish [42], while in vitro studies in motor neurons derived from SMA patients’ iPSCs showed a reduction of NRXN2 expression compared to controls, while an overexpression of NRXN2 improved the SMA motor neuron survival, and increase neurite length, suggesting it might act as a gene modifier in SMA [43].

Therefore, considering the role that CNTN1 and NRXN3 play in the nervous system, we foresee their potential as prognostic biomarkers to distinguish severe SMA1 from milder phenotypes in the context of newborn screening programs, especially in the management of asymptomatic patients.A moderate correlation was found between these markers and the age at baseline, so that future studies to ensure the robustness of our results should focus on larger cohorts with a more uniform age distributions.

To translate our findings into clinical applications, targeted assays such as Enzyme-Linked Immuno Assay (ELISA) for the identified proteins, validation in independent cohorts, and evaluation in more accessible samples like serum are underway.

In the second part of the study, we showed that, at T302, nusinersen significantly modulates the CSF proteome. Our aim was to provide insights about nusinersen-dependent protein changes at the biological processes level, rather than focusing on individual differentially expressed proteins. Through Gene ontology (GO) analysis of biological processes, we found that nusinersen causes both a common effect in all types of SMA, regardless of the severity, and specific effects in the different forms.

Specifically, nusinersen modulated biological processes related to axonogenesis and axon development in all SMA patients. SMN1 defects have been shown to significantly affect motor neuron axon development, maturation, and function in type 1 SMA patients and in a mouse model of severe SMA [36]. A marked delay of SMA motor neuron axon radial growth, which prevents a correct interaction with Schwann cells, have been demonstrated and associated with axon degeneration and loss after birth [36]. As a result, indirect markers of axon degeneration are released by suffering motor neuron and detectable in CSF and bloodstream, such as phosphorylated neurofilament heavy chain (pNfH) and neurofilament light chain (NfL), which are increased both in pre-symptomatic SMA patients with 2 SMN2 copies and symptomatic SMA patients [10, 44–46].

Our findings indicate that nusinersen modulates axonogenesis processes, in line with recent electrophysiological studies demonstrating partial enhancements in axonal maturation in SMA patients undergoing nusinersen treatment [47, 48]. Currently, no biochemical biomarkers capturing a reduction in axon deterioration upon nusinersen treatment are available, except for neurofilaments, which dosage, however, has produced discordant results [10, 45, 46, 49–52].

Among the differentially expressed proteins belonging to the axonogenesis GO term, we detected components of the semaphorine family, in particular SEMA4B, SEMA6A, SEMA7A which weredownregulated by nusinersen treatment in all patients. These results are consistent with recent untargeted proteomic studies that identified a reduction in SEMA6A and SEMA7A levels in the CSF of SMA3 patients after 22 months of nusinersen treatment [15], as well as the downregulation of SEMA7A in responders SMA3 subjects after 10 months of nusinersen [11]. Our data, combined with the previous ones, suggest semaforines, especially SEMA6A and SEMA7A, as a new class of biomarkers for monitoring nusinersen treatment in all SMA patients, regardless of their severity, reflecting axon maintenance and remodeling, as already emerged in other condition [53].

Beyond axonogenesis, nusinersen showed modulation of bioenergetic pathways in SMA1 and inflammatory pathways in SMA2 and SMA3. Previously, Errico et al. reported nusinersen affecting the glucose metabolism selectively in the CSF of SMA1 patients through NMR-metabolomic analysis [13]. Here, we confirm and strengthen these results at a protein level, showing a nusinersen-mediated boost in the protein expression of glycolytic enzymes such as PKM, ENO1, ALDOC, and PGAM1. SMA1 patient’s reduced tolerance to fasting is well known [54–56], and an imbalance in glucose metabolism has been reported in SMA2 [57]. Our results suggest a correction of glucose metabolism by nusinersen, although additional studies on blood samples will be necessary to understand whether this effect is specific to the CSF or systemic.

Moreover, with regard to the effects of nusinersen in SMA2 and SMA3 proteome, we report an increase in proteins belonging to the coagulation and the complement cascades, highly interconnected processes [58, 59], sharing common ancestral pathways [60], and contributing to a complex inflammatory network [61]. Furthermore, mediators of the coagulation cascade act as inhibitors/activators of the complement cascade, and vice versa [59]. In our results, SERPING1, an inhibitor of the classical complement cascade, was upregulated in both SMA2 and SMA3 at T302. In SMA2, an upregulation of F12, a potent activator of the C1q molecule in the classical pathway, was noted, while SMA3 showed an increase of several complement cascade components such as C1q, C1s, C2, C3, C4A, C6 and C7. Recently, Faravelli et al. reported that members of the innate immune complement pathways, including SERPING1, were among the most abundant proteins in the CSF of SMA3 patients as detected by untargeted LC-MS [15]. These findings align with recent data suggesting that the complement cascade activation participates to neuroinflammatory processes and contributes to diseases pathogenesis in various neurodegenerative disorders, including Parkinson’s, Alzheimer’s and Huntington’s diseases, Amyotrophic Lateral Sclerosis and SMA [62–65]. As such, different clinical trials investigating complement therapeutic targets are ongoing in neurologic disorders [65]. Moreover, in a pre-clinical SMA model, the aberrant upregulation of the classical complement proteins C1q and C3, was associated with dysfunction and selective elimination of synapses, and an in vivo treatment with an anti-C1q antibody induced a rescue of synapses [64].

In addition to complement activation, increased inflammatory mediators have been reported in both CSF and serum in preclinical models and in SMA patients, indicating that immune dysfunction and neuroinflammation processes are involved in the pathogenesis of SMA [38, 66–69]. In this context, we noted a decreased expression of inflammatory markers, including communication network factor 3 (NOV/CCN3), heat shock proteins HSPA1A/HSPA1B, and fibroblast growth factor receptor 2 (FGFR2), at T302 in SMA1 Responders, suggesting that treatment with nusinersen promotes better control of pro-inflammatory processes.

In summary, this untargeted LC-MS proteomic profiling in the CSF of SMA patients revealed differences in protein expression in naïve patients and showed nusinersen-related modulation in several biological processes after 10 months of treatment.

We acknowledge some limitations of the study, including the absence of CSF samples from healthy controls and untreated SMA patients, the relatively small sample size within each SMA subtype, the lack of an assay of widely used biomarkers such as NfL to parallel the results obtained, and the limited follow-up period of 10 months, that may constrain the robustness of the conclusions. Further validation in larger, independent cohorts, with uniform age distributions and long-term monitoring, is crucial to confirm the prognostic value of the identified biomarkers and better understand the duration of the observed treatment effects.

Nevertheless, despite these limitations, the integration of proteomics and machine learning in a large cohort of patients has enabled the identification of novel prognostic CSF protein biomarkers for disease severity stratification, providing a new strategy to help guiding precision therapeutic treatment.

Additional confirmatory studies on CSF and serum samples from other SMA patients and healthy controls, even after a longer treatment period, are needed to validate these results.

## Methods

### Patients characteristics

Genetically confirmed SMA patients treated with nusinersen, have been enrolled in the study from 4 tertiary Italian neuromuscular centers (IRCCS Gaslini Institute-Genoa, Nemo Center-Milan, Ospedale Bambino Gesù-Rome, and Policlinico G. Martino-Messina). SMN2 copy numbers were also available for all but one patient.

All SMA1 patients participated in the compassionate Expanded Access Program (EAP) [70], while SMA2 and SMA3 patients received treatment in a real-world setting. Nusinersen was administered following the standard protocol.

On the day of nusinersen administration, anthropometric measures, information on ventilatory support (need for non-invasive ventilation or tracheostomy), and nutritional route (oral, nasogastric tube, or gastrostomy) were noted, and functional outcome measures were performed by trained physical therapists at each site (Table 1). SMA1 patients were scored by the Children’s Hospital of Philadelphia Infant Test of Neuromuscular Disorders (CHOP INTEND) scale [71], while SMA2 and SMA3 patients were evaluated through the Hammersmith Functional Motor Scale – Expanded (HFMSE) [72]. Patients were categorized into Responders (R) and non-Responders (non-R) based on changes in motor function scores from T0 to T302, following previously described criteria. Specifically, SMA1 patients showing an increase in the CHOP score by 4 points, and SMA2-SMA3 subjects achieving a 3-point improvement on the HFMSE scale at T302 were defined Responders [73, 74].

The study was conducted in accordance with the Declaration of Helsinki and ICH GCP guidelines. All subjects and guardians provided written informed consent for the analysis of biological samples, following the approval of local ethics committees (Prot. SMALiQ_2022, 503/2022 - id 12319).

### Sample preparation and proteomic analysis

On the day of administration, CSF samples were collected after a fasting period of 4–6 h and stored at -80 °C at each site. For the purposes of this study, only CSF collected at baseline (T0; before the first nusinersen intrathecal administration) and at day 302 (T302; before the sixth infusion), were centralized at IRCCS Gaslini, and subsequently subjected to proteomic analysis.

One hundred ul of CSF were denatured, reduced and alkylated in 100 ul of iST-LYSE buffer (PreOmics) for 10 min at 95 °C, 1000 rpm. Proteins were isolated by PAC method [75]. Briefly, protein aggregation was induced by addition of 70% ACN and 200 ug of magnetic beads were added to capture aggregated proteins. Magnetic beads were retained by magnet and the supernatant was removed. Beads were washed one time with 1 ml acetonitrile, followed by two wash with 1 ml 70% ethanol. Washed beads were resuspended in 100 ul TRIS 25 mM pH 8 and captured proteins were digested O.N. at 37 °C with 0.7 ug Trypsin and 0.3 ugLysC. Obtained peptides were desalted in Stage-Tips [76] and analyzed by a nano-Ultra High Performance Liquid Chromatography-Tandem Mass Spectrometry system (nano-UHPLC-MS/MS) using an Ultimate 3000 RSLC coupled to an Orbitrap Fusion Tribrid mass spectrometer.

Elution was performed with an EASY spray column (75 μm x 50 cm, 2 μm particle size, Thermo Scientific) at a flow rate of 250 nl/min with a 100 min non-linear gradient consisting of an increase from 7 to 27% solution B (80% ACN and 20% H2O, 5% DMSO, 0.1% FA) in 57 min, with a further increase to 45% B in 15 min, followed by1-minute wash at 80% B and a 20 min re-equilibration at 2% B.

MS scans were acquired at a resolution of 120,000 between 375 and 1,500 m/z and an AGC target of 4.0E5, 50 ms maximum injection time. Advanced Peack Determination was enabled for MS1 measurements. MS/MS spectra were acquired in the linear ion trap (rapid scan mode) with an AGC target of 3.0E4 and a 30 ms maximum injection time. For precursor selection, were prioritized the least abundant signals in the three ranges 375–575 m/z, 574–775 m/z and 774–1500 m/z. Quadrupole isolation with a 0.7 m/z isolation window was used, and Dynamic Exclusion was set at 25 s. HCD was performed using 30% collision energy.

MaxQuant software version 1.6.17.0 was used to process data [77]. A false discovery rate was set at 0.01 for the identification of proteins, peptides and peptide-spectrum match (PSM). A minimum of 6 amino acids was required for peptide identification. Andromeda engine, incorporated into MaxQuant software, was used to search MS/MS spectra against Uniprot human database (release UP000005640_9606 December 2020). In the processing the variable modifications are Acetyl (Protein N-Term), Oxidation (M), Deamidation (NQ), on the contrary, the Carbamidomethyl (C) was selected as fixed modification. Quantification intensities were calculated by the default fast MaxLFQ algorithm with the activated option ‘match between runs’.

### Machine learning algorithm

The Random Forest (RF) machine learning algorithm, previously applied to metabolomics and proteomics data for classification and regression analysis [78, 79], was adopted to classify SMA types based on protein expression levels at baseline. Random Forest models were trained for classification of SMA subtypes using the proteomics data obtained at T0. All steps were carried in “R” free software using “caret” (ver. 6.0–94) and “randomForest” (ver. 4.7–1.1) packages. The detailed strategies employed during optimization and prevention of overfit and data leakage are described in the Supplementary Methods. All the steps were carried out individually, with proteomics data only. To investigate the effects of different hyperparameter tuning strategies on obtained classification accuracies, data partitioning ratio, mtry (number of features to be randomly selected at each split), number of trees, and maximum number of nodes were assigned to different sets of values. Besides varying sets of parameters, addition of cross-validation and post-processing (normalization, transformation, scaling) of training and test data after partitioning were included in optimizations to avoid potential data loss. At the end of optimizations, normalized and scaled T0 proteomics data were used, and partitioned into train and test datasets in 80% and 20% ratios, respectively. Model training was carried out using 1000 trees and maximum number of nodes, set to 3. Classification accuracy of the trained model on test data was calculated by comparing the real and predicted SMA classes. List of important variables were recorded for the trained model by extracting top 30 features sorted by mean decrease in Gini coefficient. Each step, ranging from data partitioning to the selection of important variables, was repeated 100 times, with the seed set to numbers between 1 and 100. Finally, a set of features common in all lists of top 30 important variables from 100 trained models was obtained.

### Statistical analysis

Log2-transformed and median-centered intensity values were used for downstream statistical analyses. PERMANOVA with Euclidean distance method was used to test significance of subtype, sex, age, smn2 gene copy number, and BMI. For comparison of protein compositions of SMA1, SMA2, and SMA3 subtype samples before treatment, analysis of covariance (ANCOVA) was used by adjusting for age. Features with raw p value less than 0.05 were evaluated as significant and used in clustering for each pairwise comparison. Before and after treatment comparisons of SMA1, SMA2, and SMA3 patients were carried out individually, using paired two-sided t-test, and obtained p values were adjusted for multiple hypothesis testing using Benjamini-Hochberg method. Log2 fold changes in responders and non-responders were calculated by subtracting the values at T302 from T0 for each SMA subtype, and unpaired two-sided t-test was used for the comparison. Raw p values were used for significance. For each pairwise comparison results, volcano plots were produced using EnhancedVolcano (ver. 1.16.0). Principal component analysis (PCA) was carried out using FactoMineR (ver. 2.9), and visualizations with different pairs of dimensions were obtained using factoextra package (ver. 1.0.7).

For correlation analysis between age and 9 biomarker candidates, Spearman correlation was performed and obtained p values were adjusted using Benjamini-Hochberg method.

#### Hierarchical clustering

Intensity data of the filtered features were scaled to have a mean value of zero with standard deviation of one. The Euclidian distances were calculated between samples and between proteins using dist function, and then, Ward-D2 clustering was performed for samples and for proteins by hclust function. Heatmaps were plotted via heatmap.2 function in gplots package (ver. 3.1.3). In order to divide clustered into two clusters, mainly corresponding to increasing and decreasing proteins for each pairwise comparison, cutree function with k = 2 was used.

#### Pathway enrichment

Gene Ontology (GO) enrichment analysis was performed for proteins in each cluster, separately. For that purpose, respective gene symbols of the proteins in each cluster were converted into Entrez ID’s by mapIds function in AnnotationDbi package org.Hs.eg.db (ver. 3.14.0). If Entrez ID could not be obtained for a protein, and additional manual curation step was performed using protein ID as query on UniProt website (www.uniprot.org/uniprotkb/< protein.id>/entry, 10.1093/nar/gkh131). If the protein IDs for a single protein entry correspond to different proteins, it was excluded from enrichment analysis. For each clusters, obtained list of Entrez IDs were submitted to ShinyGO (ver 0.76, http://bioinformatics.sdstate.edu/go/, 10.1093/bioinformatics/btz931) after selecting “Human” as the organism with other default settings. Enrichment was performed using three different GO pathway databases; “GO Biological Process”, “GO Cellular Compartment”, and “GO Molecular Function”. Tables for all enriched pathways and lollipop plots were exported.

In addition to the GO enrichment analysis, the Kyoto Encyclopedia of Genes and Genomes (KEGG) enrichment analysis of biological processes (BP) was performed. The results are provided as Supplementary Fig. 2a-f.

## Electronic supplementary material

Below is the link to the electronic supplementary material.

Supplementary Material 1

Supplementary Material 2

Supplementary Material 3

Supplementary Material 4

Supplementary Material 5

## Data Availability

The mass spectrometry data have been deposited to the ProteomeX-change Consortium via the PRIDE partner repository with the dataset identifiers: PXD047529 (Reviewer account details: Username: reviewer_pxd047529@ebi.ac.uk Password: oRxG3oEc).
