# Supplementary figures and images for: Proteomics profiling and machine learning in nusinersen-treated patients with spinal muscular atrophy
Source: Cell Mol Life Sci. 2024 Sep 10;81(1):393. doi: 10.1007/s00018-024-05426-6 (PMC11387582; doi:10.1007/s00018-024-05426-6)

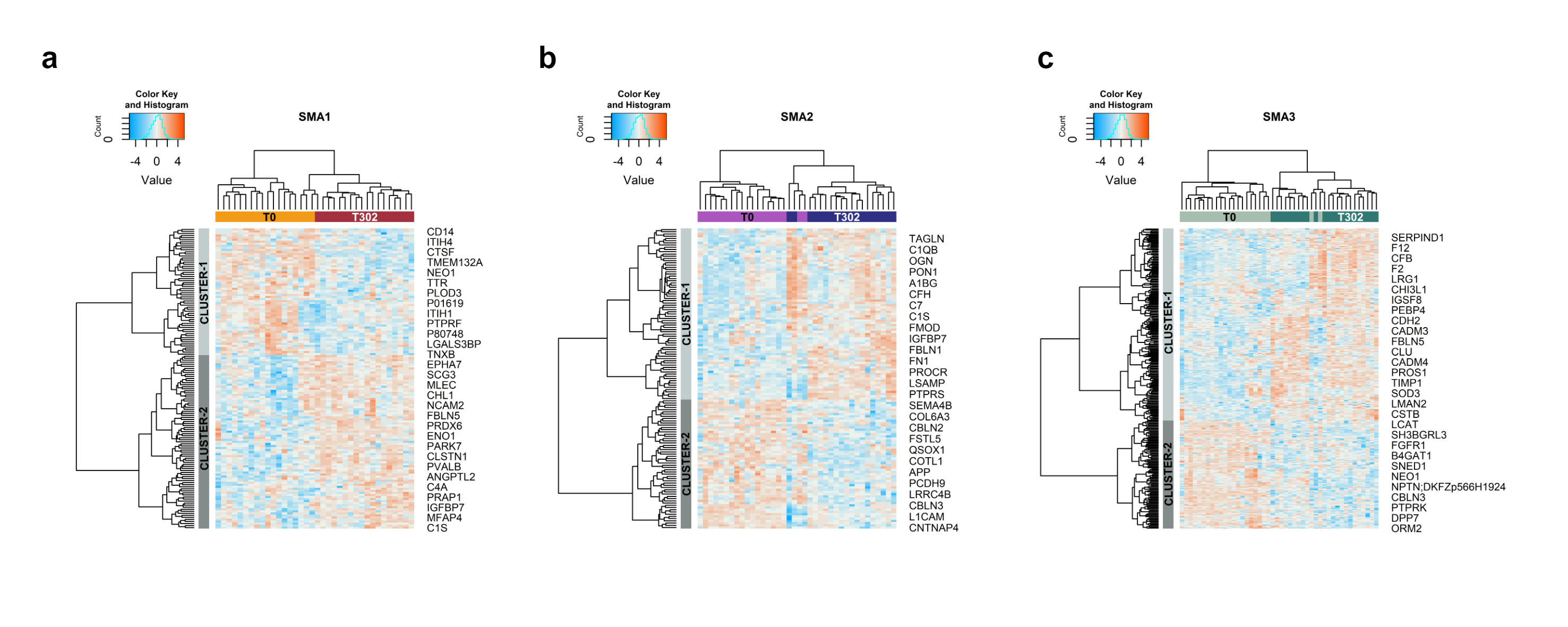

Supplement: Supplementary file 2 — Supplementary Material 2 [file 18_2024_5426_MOESM2_ESM.png]

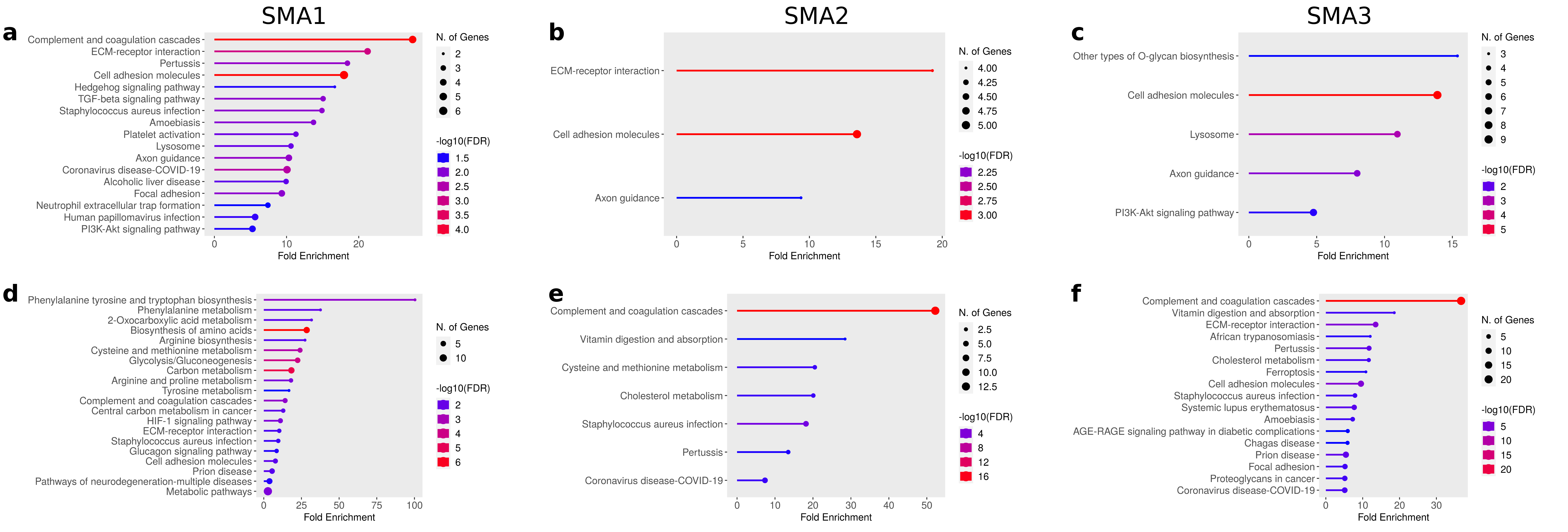

Supplement: Supplementary file 4 — Supplementary Material 4 [file 18_2024_5426_MOESM4_ESM.png]
