# Supplementary material for: Proteomics profiling and machine learning in nusinersen-treated patients with spinal muscular atrophy
Source: Cell Mol Life Sci. 2024 Sep 10;81(1):393. doi: 10.1007/s00018-024-05426-6 (PMC11387582; doi:10.1007/s00018-024-05426-6)
Supplement: Supplementary file 3 — Supplementary Material 3 [file 18_2024_5426_MOESM3_ESM.docx]

**Machine learning methods should be described in more detail, especially regarding the possibility of overfitting.**

The methods to reduce risk of overfitting in the trained model were described in detail below:

- **Hyperparameter tuning and cross validation:** “mtry” is the number of features (proteins) randomly selected among the features in original dataset to use in each splitting. By default, the algorithm uses mtry value equals to square root of total number of features. Another term is “ntree” which stands for the number of trees used in each model training to have final decision based on the majority. Last parameter is the number of “maxnodes” which is maximun number of terminal node to determine the complexity of the tree. We applied hyperparameter tuning by applying different parameter settings during model training using train dataset, which will select the best optimum model with highest performance at the end. In addition, 10-fold cross validation with 3 repeats were applied. None of the attempts by testing different combinations of the parameters changed the accuracy values.
- **Boruta feature selection:** Feature selection before random forest model training was performed using another random forest-based method, called Boruta. It helps to remove irrelevant features (proteins) that may not be significant in classification problem and helps to improve the model training performance. It adds shadow features to the datasets and perform random forest algorithm for classification, then removes the features in original dataset that shows less significance than the artificial shadow features. When we filtered proteins using Boruta algorithm and fed the filtered dataset to the random forest algorithm, we saw that neither training dataset accuracy, nor the trained model performance on test dataset was changed for all the 100 independent models and all the models have accuracy values of 1.0.
- **Removing highly correlated proteins:** Another tested approach was removal of highly correlated proteins to decrease redundancy and reducing risk of overfitting in trained model performance. For that purpose, pairwise correlation between each protein was measures, and the proteins with correlation coefficient equal or higher than absolute value of 0.9 were removed prior dataset splitting and model training, however, all the models have accuracy of 1.0 for both trained and test datasets.
- **Individual data normalization and scaling for train and test datasets:** To prevent any chance of data leakage that might cause test data prediction accuracy of 1.0, non-normalized and non-scaled proteomics data were split into train and test datasets, and normalization and scaling procedures were applied on train and test datasets individually. When random forest model trainings with same settings were performed on train dataset, all 100 independent models had accuracy score of 1.0 on test dataset.
