# Supplementary material for: Proteomics profiling and machine learning in nusinersen-treated patients with spinal muscular atrophy
Source: Cell Mol Life Sci. 2024 Sep 10;81(1):393. doi: 10.1007/s00018-024-05426-6 (PMC11387582; doi:10.1007/s00018-024-05426-6)
Supplement: Supplementary file 5 — Supplementary Material 5 [file 18_2024_5426_MOESM5_ESM.docx]

**Supplementary Table 1.** The results of the 100 Random Forest models are provided in the table (sheet 1). Correlation analysis between age and the 9 biomarker candidates was performed by Spearman correlation, and obtained p values were adjusted using Benjamini-Hochberg method (sheet 2).

**Supplementary Fig. 1.** Hierarchical clustering of the samples belonging to (a) SMA1, (b) SMA2, and (c) SMA3 type groups based on the significantly differentially expressed proteins (DEPs) obtained from comparison of T0 and T302 protein levels. Samples are clustered on x axis, and proteins were clustered in y axis based on the abundance profiles. Low and high abundance of proteins were shown by blue and orange color shades. Proteins were divided into two clusters based on their abundance at each time points.

**Supplementary Fig. 2.** Top 20 KEGG pathways for biological processes (BP) enriched by mainly down-regulated (a-c) and up regulated DEPs (d-f), obtained from two clusters from each heatmap, are shown by lollipop plots for each SMA type. Fold enrichment represents the ratio of the percentage of matched proteins in the query list with pathway-associated proteins to the percentage of query with the background. Bar colors are corresponding to the false discovery rate (FDR) corrected p values for each enriched pathway, and the size of dots are proportional to the number of proteins associated with the respective pathway.
